# Advances in Forensic Entomotoxicology for Decomposed Corpses: A Review

**DOI:** 10.3390/insects16070744

**Published:** 2025-07-21

**Authors:** Sen Hou, Zengjia Liu, Jiali Su, Zeyu Yang, Zhongjiang Wang, Xinyi Yao, Zhou Lyu, Yang Xia, Shuguang Zhang, Wen Cui, Yequan Wang, Lipin Ren

**Affiliations:** 1 School of Forensic Medicine, Jining Medical University, Jining 272067, China; housen731@163.com (S.H.); lzjia001@163.com (Z.L.); yongyuanshishang@163.com (J.S.); yangzy409@126.com (Z.Y.); wzjztzj626@126.com (Z.W.); yaoxinyi04171@126.com (X.Y.); cuiwenmdd@163.com (W.C.); 2 Criminal Investigation School, Southwest University of Political Science & Law, Chongqing 401120, China; forensicluzhou@hotmail.com; 3 Department of Forensic Science, School of Basic Medical Sciences, Central South University, Changsha 410017, China; csu_xiayang@163.com; 4 Department of Forensic Science, School of Basic Medical Sciences, Inner Mongolia Medical University, Hohhot 010107, China; shuguang_cf@163.com

**Keywords:** forensic entomotoxicology, xenobiotic exposure, decomposed corpses, multi-omics, machine learning

## Abstract

Forensic entomotoxicology is a subdiscipline that utilizes necrophagous insects as bioindicators for detecting drugs and toxicants in decomposed remains, particularly in cases where conventional biological matrices are no longer available. Toxic substances can profoundly alter insect development, physiology, and community succession, potentially impacting the accuracy of postmortem interval (PMI) estimation. This review systematically summarizes the effects of various xenobiotics, including pesticides, illicit drugs, sedatives, heavy metals, and antibiotics on larval growth, physiological traits, and gut microbial composition in forensically relevant flies. However, most studies to date have relied primarily on phenotypic observations, with limited insight into underlying molecular mechanisms. Significant interspecies and dose-dependent variability also exists in the absorption, metabolism, and physiological responses to xenobiotics. We highlight recent advances in multi-omics technologies that facilitate the identification of molecular biomarkers associated with xenobiotic exposure, particularly within the insect detoxification system. Key components such as *cytochrome P450 monooxygenases* (*P450s*), *glutathione S-transferases* (*GSTs*), and *ATP-binding cassette* (*ABC*) transporters play essential roles in xenobiotic metabolism and insecticide resistance. Additionally, the insect fat body serves as a central hub for detoxification, hormonal regulation, and energy metabolism. It integrates signals related to xenobiotic exposure and modulates larval development, making it a promising model for future mechanistic studies in insect toxicology. Altogether, this review offers a comprehensive and reliable framework for understanding the complex interactions between toxic substance exposure, insect ecology, and decomposition in forensic investigations.

## Simple Summary

Forensic entomotoxicology is a specialized subfield that investigates how insects feeding on decomposed remains can serve as bioindicators for the detection of drugs and toxicants, particularly in cases where traditional samples are no longer available due to advanced decomposition. Xenobiotics can markedly influence insect growth, physiology, and behavior, potentially leading to inaccuracies in estimating the postmortem interval (PMI). This review systematically explores the effects of common xenobiotics, including pesticides, illicit drugs, sedatives, heavy metals, and antibiotics on insect physiological traits and gut microbiota. We also highlight recent advances in multi-omics technologies that reveal how insects absorb, metabolize, and respond to these substances. Key detoxification enzymes, including *cytochrome P450s*, *glutathione S-transferases* (*GSTs*), and *ABC* transporters, play crucial roles in xenobiotic metabolism and resistance, with the insect fat body acting as a major organ for detoxification, hormonal regulation, and energy metabolism. Overall, this review provides new insights into the advancement of forensic entomotoxicology and highlights promising avenues for improving the reliability of insect-derived evidence in forensic investigations.

## 1. Introduction

In cases of advanced decomposition, particularly when remains are skeletonized, traditional toxicological methods often become unfeasible due to tissue degradation [1,2,3]. In such scenarios, necrophagous insects that colonize decomposing remains serve as valuable alternative toxicological samples [4]. This approach, known as forensic entomotoxicology, involves two primary objectives: first, the detection of toxic substances accumulated in necrophagous insects to aid in determining the cause of death in decomposed remains; moreover, the investigation of how these substances affect insect developmental biology, which is essential for improving the accuracy of PMI estimation by accounting for xenobiotic-induced deviations in insect growth patterns [5]. Insects typically acquire xenobiotics through ingestion of decomposing tissues and absorption of body fluids from the corpse [6]. These substances can significantly alter the normal developmental cycle of necrophagous larvae. For example, opioids and certain antidepressants have been shown to delay the larval development of blowflies [7,8], whereas stimulants such as cocaine and methamphetamine may accelerate growth, potentially leading to premature pupation and adult emergence [9]. If these pharmacological effects on insect development are not taken into account, PMI estimation may be substantially miscalculated, potentially undermining the accuracy of forensic investigations [10]. Therefore, it is essential to construct integrated and dynamic models that encompass the complex interactions among xenobiotics, insect physiology, and environmental variables, which collectively influence decomposition processes and insect life history traits. These models will enhance the scientific robustness and reliability of entomotoxicological data in forensic investigations.

Xenobiotics can modulate insect development primarily by activating the insect detoxification system, which includes a coordinated network of metabolic enzymes and transporters [11]. However, the efficiency of xenobiotic absorption, metabolism, and excretion varies significantly across different species; this variation is primarily driven by the enzyme systems. *Cytochrome P450 monooxygenases* (*P450s*) reduce the biological activity of toxic compounds through oxidative biotransformation [11,12]. *P450s* are widely regarded as the primary enzymes involved in detoxification and are strongly associated with resistance to a broad spectrum of synthetic insecticides. Notably, many *P450s* that respond to insecticides are also inducible by phytochemicals, reflecting their evolutionary versatility [13]. Following initial modification, *glutathione S-transferases* (*GSTs*) and *UDP-glucuronosyltransferases* (*UGTs*) further conjugate these metabolites to enhance their solubility and facilitate elimination [14]. These conjugated products are subsequently exported from cells by *ATP-binding cassette* (*ABC*) transporters [15]. This system is highly adaptable, and its gene expression can be upregulated in response to xenobiotic exposure, contributing to insecticide resistance [16]. Furthermore, the insect fat body, functionally analogous to the vertebrate liver and adipose tissue, serves as the primary site for detoxification enzyme activity. In addition to its role in xenobiotic metabolism, it is essential for nutrient storage, energy homeostasis, immune responses, and endocrine regulation [17]. Fat body cells undergo mitosis during embryogenesis, endoreplication during larval development, and significant structural remodeling during metamorphosis [18]. Recent studies highlight their central role in integrating hormonal and nutritional signals that coordinate larval growth, lifespan regulation, and feeding behavior [19], making the fat body cell a target organ between detoxification and insect physiology.

As mentioned above, the physiological and biochemical alterations induced by xenobiotic exposure serve as valuable biomarkers in forensic entomotoxicology, a field with significant potential for forensic applications [11]. However, interpreting toxicological data derived from insect samples remains challenging. In particular, species-specific variability in xenobiotic absorption, distribution, and metabolism can lead to inconsistent bioaccumulation patterns, complicating comparisons across different insect taxa and potentially limiting the reliability of toxicological data [20,21,22]. Additionally, environmental factors, such as temperature, humidity, and the presence of other chemical substances, can further influence both insect colonization behavior and xenobiotic absorption, thereby altering the toxicological profile detected in insect tissues [23,24]. Furthermore, there is a lack of standardized protocols for the collection, preservation, and analysis of entomological specimens, which hampers consistency across casework and reduces reproducibility [25]. Despite these limitations, recent technological advancements are expanding the utility of forensic entomotoxicology. Multi-omics approaches, including mass spectrometry-based metabolomics and high-throughput sequencing, now enable the sensitive detection and quantification of a broad range of xenobiotics in insect tissues [26]. For instance, metabolomic profiling can detect subtle biochemical alterations induced by xenobiotic exposure, serving as indirect indicators of drug ingestion or poisoning [27]. Furthermore, recent studies have revealed that xenobiotics can alter microbial succession patterns, which, in turn, influence insect colonization dynamics and decomposition rates [28].

**Figure 1:**
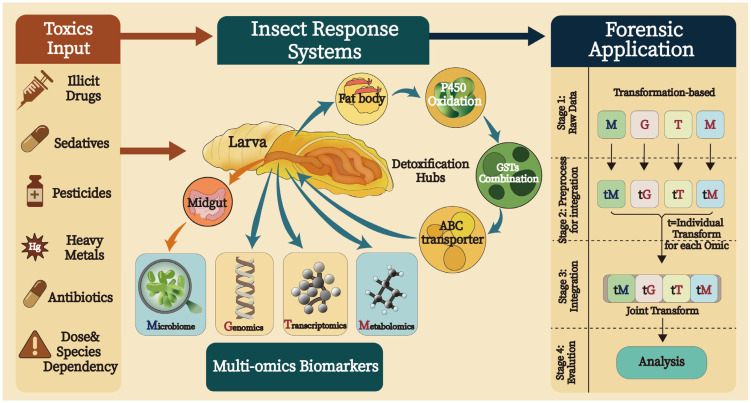
Integrated multi-omics framework for insect-mediated toxicological assessment and forensic application. This schematic illustrates a multi-omics strategy for detecting and interpreting xenobiotic exposure in necrophagous insects. Xenobiotics such as drugs, pesticides, heavy metals, and antibiotics enter insect larvae via ingestion of decomposing tissues, primarily affecting the midgut and fat body, which are key sites for detoxification and metabolism. Exposure triggers coordinated detoxification pathways, including *cytochrome P450* oxidation, *GST*- and *UGT*-mediated conjugation, and *ABC* transporters, leading to changes across multiple biological layers. These omics data are further processed for forensic application through a four-stage pipeline. This framework accounts for species-specific and dose-dependent variability, offering a robust tool for forensic investigations where conventional matrices are unavailable.

This review aims to provide a comprehensive understanding of how specific xenobiotics influence carrion community succession, the development of necrophagous insects, and their underlying physiological and biochemical responses, which provides valuable insights for improving the accuracy of PMI estimation and determining the cause of death, particularly in cases involving advanced decomposition and suspected poisoning [29]. In this scenario, we integrate multi-omics technologies, including transcriptomics, and mass spectrometry-based metabolomics, as well as the emerging role of deep learning algorithms (Figure 1). These techniques also provide insights into how xenobiotics modulate insect gene expression, metabolism, and developmental physiology [30]. In particular, omics-based approaches enable the identification of molecular biomarkers that reflect insect exposure to various xenobiotics such as opioids, stimulants, pesticides, and heavy metals, thereby enhancing the interpretative value of entomological evidence in forensic investigations. Simultaneously, deep learning is being developed to model the complex and nonlinear relationships between xenobiotic concentration, insect developmental rates, environmental variables, and microbial community dynamics [31]. These models hold great promise for adjusting PMI estimations by accounting for xenobiotic-induced delays or accelerations in insect development. Ultimately, the integration of ecological, physiological, and computational frameworks is transforming forensic entomotoxicology into a robust, interdisciplinary field capable of addressing complex forensic challenges.

## 2. Retrospective Analysis of Forensic Entomotoxicology

Determining the PMI and cause of death in cases involving advanced decomposition remains a major challenge in forensic investigations. As a corpse decays, it undergoes a series of complex biological, chemical, and microbial changes that can degrade or obscure critical forensic evidence. Putrefied tissues are often unsuitable for conventional toxicological analyses due to the loss of viable biological matrices [5,25]. This limitation is especially acute in cases of drug-related deaths occurring in remote or delayed-discovery scenarios [6]. In such scenarios, forensic entomotoxicology has emerged as a valuable alternative tool, enabling the detection of drugs and toxicants by analyzing necrophagous insects [32,33]. Numerous studies have validated it, highlighting the capacity to bioaccumulate drugs and other xenobiotics from corpses even when traditional samples are no longer available [34,35]. Moreover, xenobiotics accumulation has been shown to affect key physiological parameters in insects, including body size, weight, developmental duration, and survival rate (Table 1). These physiological disruptions can subsequently influence the succession patterns and colonization behavior of necrophagous species, thereby affecting decomposition dynamics and potentially leading to inaccuracy in PMI estimation (Table 2). Thus, it is critical to standardize entomotoxicological protocols, which would not only enhance the validity of PMI estimation but also contribute to meet the Daubert criteria for the admissibility of expert evidence in court [36].

### 2.1. Impact of Pesticides on Succession Patterns and Development of Necrophagous Flies

Pesticide ingestion remains one of the most common methods of suicide worldwide, with organophosphates and carbamates being among the most frequently implicated agents in both accidental and intentional poisoning cases [66]. The residue of pesticides in corpses can significantly influence larval development and insect succession patterns, thereby compromising the accuracy of PMI estimation [44,67]. Many of these pesticides act as neurotoxins by inhibiting acetylcholinesterase, a key enzyme responsible for the breakdown of neurotransmitters, ultimately disrupting normal neural function [68]. The sensitivity of necrophagous fly species to pesticide exposure varies considerably. For example, Cavalcante et al. [58] evaluated the effects of diazinon (organophosphate) on the composition and succession of calliphorid species in the tropical savannas of the Amazon, which indicated that diazinon exposure slowed down the decomposition stages of carcasses, and significantly reduced the abundance of immature calliphorids at higher concentrations. Similarly, Jales et al. [44] observed that *Lucilia eximia* larvae displayed increased activity and more frequent movements and lateral contractions when exposed to intermediate and high doses of terbufos. Conversely, *Peckia chrysostoma* was negatively affected, suggesting species-specific differences in mobility responses to pesticide exposure [44]. Moreover, a previous study reported that a higher dose of terbufos accelerated carcass decomposition within 24 h, decreased the species richness and larval abundance, and delayed the arrival of necrophagous species [66]. Additionally, Bhardwaj et al. [69] investigated the effect of aluminum phosphide (AlP) on the larval morphometry and life cycles of *Chrysomya megacephala* and *Chrysomya rufifacies*, showing that AlP exposure significantly increased larval length and weight, accelerating development up to pupation at higher concentrations. Indeed, pesticides induce oxidative stress and lipid peroxidation, making the assessment of peroxidation and enzymatic activity crucial for evaluating toxin-induced stress in insects [70]. These findings highlight the complex biochemical and physiological impacts of pesticide exposure on necrophagous insects, which must be considered in forensic entomology, as it can significantly alter developmental time and lead to inaccurate PMI estimations in forensic investigations.

### 2.2. Effect of Psychoactive Drugs on the Development of Necrophagous Flies

As we all know, the abuse of illicit drugs presents significant global public health and socio-economic challenges, contributing to over 200,000 deaths worldwide [71], which is therefore highly relevant to entomotoxicological studies. Stimulants (e.g., cocaine, methamphetamine) have been shown to enhance metabolic activity in necrophagous insects, often resulting in accelerated development and altered life history traits [8,33,38]. For example, Wood et al. [9] demonstrated that cocaine exposure reduced the larval length and weight of *Calliphora vomitoria*, shortened pupation time, and accelerated eclosion, suggesting increased metabolic activity. Similarly, Mullany et al. [8] found that methamphetamine exposure in *Calliphora stygia* significantly accelerated larval growth and increased body size across life stages, with pupae exhibiting extended developmental duration up to 78 h longer than the control group. Goff et al. [72] also observed that the larval development of *Sarcophaga ruficornis* accelerated in response to increasing concentrations of methamphetamine. While the overall pupation time was extended by up to 48 h compared to controls, larvae exposed to the highest concentrations were significantly shorter in length, indicating dose-dependent effects.

In contrast, central nervous system depressants (e.g., heroin and morphine) inhibit neural activity and suppress metabolic function, typically resulting in prolonged development times of flies and delayed pupation [9,73,74]. For instance, heroin appeared to delay the decomposition process of rabbit carcasses but had no significant impact on insect succession patterns [63]. In this scenario, it is therefore essential to more comprehensively understand the effects of illicit drugs on the development of necrophagous flies. If the effects of stimulants or depressants are not adequately considered, inaccurate PMI estimation may occur. In addition, sedatives have also been implicated in numerous overdose-related suicides worldwide. Recent studies have shown that the developmental rate of *S. ruficornis*, *C. megacephala*, *Chrysomya saffranea*, and *C. rufifacies* was delayed with increasing concentrations of zolpidem tartrate and lorazepam; this was accompanied by significant reductions in morphological parameters such as length and weight [47,48,49]. These findings underscore the complex, dose-dependent, and species-specific responses of necrophagous flies to pharmacological agents. Given the increasing prevalence of poly-drug use in forensic investigations, it is essential to account for the potential interactions and cumulative effects of multiple substances in entomotoxicological analyses. Accordingly, we recommend that future studies define and validate appropriate concentration ranges for individual and combined drug exposures when assessing their impact on fly development to ensure more accurate interpretation of PMI.

### 2.3. Effects of Antibiotics on the Development of Necrophagous Flies

Furthermore, global antibiotic use has increased by approximately 46% since 2000 [75], and usage in the animal husbandry sector alone is projected to reach 107,000 tons by 2030 [76]. This rising trend has led to widespread overuse and the emergence of antibiotic resistance. Importantly, antibiotics can profoundly disrupt the microbial communities within decomposing remains and the necrophagous insects that colonize them [77]. Fly larvae depend on symbiotic gut bacteria to facilitate digestion and nutrient absorption; thus, antibiotic exposure can reduce the diversity and abundance of these gut microbes, impairing larval development and metabolic function [78]. For example, Preußer et al. [50,51,52] examined the effects of ceftriaxone and levofloxacin on maggot development of *Lucilia sericata*, *C. vomitoria*, and *Protophormia terraenovae*. The effects varied significantly among species. In *C. vomitoria*, levofloxacin reduced maggot size and delayed both development and pupation [51]. In *L. sericata*, high concentrations of levofloxacin significantly delayed pupation and increased mortality, although larval length and weight remained largely unaffected [50]. In contrast, in *P. terraenovae*, both ceftriaxone and levofloxacin significantly increased larval length and weight, while accelerating development and reducing pupation time [52]. More recently, Tang et al. [53] demonstrated that ciprofloxacin had no observable effect on the larval length of *Sarcophaga peregrina*, though higher concentrations accelerated larval growth rates. These findings suggest that antibiotic effects are both species-specific and dose-dependent. Therefore, we speculate that differences in dominant gut microbiota among fly species may influence how antibiotics affect larval physiology. In particular, the suppression of beneficial intestinal flora could directly impair larval development. Given these findings, it is critical to investigate the influence of antibiotics on necrophagous species; otherwise, overestimation of PMI may occur.

### 2.4. Effect of Heavy Metals on the Development of Necrophagous Flies

Environmental pollution remains one of the most pressing global challenges, with heavy metals posing particularly severe toxicological risks to both flora and fauna [79]. Insects exposed to heavy metal pollutants can bioaccumulate these contaminants, resulting in alterations to key life-history traits at the organismal level [80]. Previous studies have demonstrated that various heavy metals, including cadmium (Cd), mercury (Hg), lead (Pb), zinc (Zn), iron (Fe), copper (Cu), and cobalt (Co), negatively impact the development and survival of blowflies such as *C. vicina* [55], *C. albiceps* [81], *C. megacephala* [54], and *P. terraenovae* [82], and *L. sericata* [56]. These effects are primarily due to the disruption of enzymatic processes critical for growth, often resulting in delayed development and increased mortality. For instance, Kökdener et al. [56] reported that in *L. sericata*, the presence of Zn, Cd, and Cu shortened larval and pupal development times, while higher metal concentrations significantly reduced survival rates, larval length, and pupal weight. The results indicate that when determining the larval age based on length, if the exposure to heavy metals is ignored, the PMI can be inaccurately estimated up to 7.2–22 h. But, in contrast, Singh and Bhupinderjit [54] illustrated that cadmium chloride negatively affected all the life stages of *C. megacephala*, with developmental time increasing, and Cd concentration potentially leading to PMI errors ranging from 18 to 86 h. Additionally, exposure to heavy metals has been shown to damage the peritrophic membrane in the midgut tissue of insects, resulting in epithelial cell lesions, inhibition of essential metabolic enzymes, and developmental abnormalities [83]. These disrupt ATP production, reduce energy availability, and impair nutrient absorption and digestion, ultimately leading to decreased growth and lower survival rates [80]. Moreover, heavy metals can also induce oxidative stress, causing lipid peroxidation and further physiological damage [80,84]. Considering all of this, if the presence of heavy metals is not taken into consideration, it may lead to substantial errors when using the normal development of necrophagous insects for PMI estimation.

## 3. Importance of Multi-Omics Technologies in Entomotoxicology

Multi-omics technologies have become increasingly valuable in entomotoxicology, providing insights into the effects of toxic substances on gene expression, metabolic pathways, and gut microbial communities within necrophagous insects [30]. These approaches significantly enhance the accuracy of PMI estimation and the detection of toxic substances [26]. Transcriptomic analyses, for example, have shown that larvae exposed to high levels of toxins upregulate stress-response genes such as *heat shock proteins* (*HSPs*) and antioxidant enzymes, which are essential for mitigating toxin-induced damage [70]. However, this adaptation may divert energy and resources away from growth and development, ultimately impairing larval development [85]. For example, Li et al. [86] demonstrated that high cadmium (Cd) exposure in honeybees led to acute mortality and chronic oxidative stress, with marked downregulation of antioxidant genes (*AccSOD1*, *AccTPx3*, *AccTPx4*) and decreased superoxide dismutase (SOD) activity, which resulted in increased malondialdehyde (MDA) levels, a marker of lipid peroxidation. Similarly, immune-related genes (*AccAbaecin*, *AccApidaecin*) and acetylcholinesterase activity were suppressed under Cd exposure, further affecting the insects’ physiological resilience. In addition, metallothionein (MT) is thought to play an important role in metal homeostasis and detoxification for some insects [87]. Zhang et al. [88] identified and characterized three MT genes in the black soldier fly, RNAi results of *BSFMT2B* showed that the larval weight decreased significantly, and the mortality of larvae increased significantly. Therefore, it is necessary to further explore the physiological function of metallothionein in necrophagous insects. Subsequently, Tony et al. [89] assessed the impact of aluminum phosphide (AlP) and cypermethrin (CP) on the antioxidant enzymes in the third instar of *C. megacephala* maggots. The results indicated that maggots exposed to AlP- or CP-contaminated tissues displayed significant reductions in total protein (TP), total antioxidant capacity (TAC), SOD, glutathione S-transferase (GST), and catalase (CAT), while levels of aspartate aminotransferase (AST), alanine aminotransferase (ALT), and MDA were increased [89]. Additionally, *P450s* have been identified as key enzymes in insecticide selectivity, offering insights into their molecular functions and roles in metabolizing xenobiotics [12]. Yang et al. [90] further revealed that sublethal doses of dichlorvos in earthworms significantly inhibited *CYP1A2* and *CYP2C9*, while altering metabolic profiles, including increased glucose and ornithine levels and reductions in malate and essential amino acids such as glutamine, tryptophan, phenylalanine, and leucine.

Metabolomics has emerged as a crucial tool in forensic entomotoxicology, enabling the detection of drug metabolites in insect tissues, which can provide indirect evidence of xenobiotic exposure in decomposed remains, particularly in suspected overdose or poisoning cases [27,91]. Larvae that feed on drug-contaminated tissues can absorb and biotransform these compounds, producing specific metabolites that serve as indicators of the substances present at the time of death. For instance, Ishak et al. [92] utilized metabolomic profiling to detect heroin-related compounds in *Lucilia cuprina*, identifying key metabolites such as tryptophan, hydromorphone, and morphine, primarily in second- and third-instar larvae. The detection of these compounds in larval tissues provides strong evidence of drug ingestion by the host. Subsequently, Buratti et al. [93] developed and validated a qualitative method using ultra-high-performance liquid chromatography coupled with Fourier-transform mass spectrometry (UHPLC-TF-MS) to identify three opioids (morphine, codeine, and methadone) and their metabolites (6-monoacetylmorphine and 2-ethylidene-1,5-dimethyl-3,3-diphenylpyrrolidine) in larvae of *L. sericata*. Although the specific metabolic pathways through which larvae process these drugs remain poorly understood, the identification of such metabolites underscores the utility of metabolomics. These findings not only contribute to confirming the cause of death but also support more accurate PMI estimations by linking drug metabolism to larval developmental stages.

Microbiomics offers valuable insights into the interplay between xenobiotics and the gut microbiota of insects, revealing how these interactions influence larval growth and decomposition rates [94]. The insect gut microbiome plays a crucial role in nutrient digestion, immune modulation, and the biotransformation of xenobiotics through microbial enzymatic activity [95]. For example, Ruan et al. [78] reported that antibiotic exposure in *Hermetia illucens* (black soldier fly) larvae significantly reduced microbial diversity and altered the taxonomic composition of the gut community, characterized by a decline in *Actinobacteriota* and an increase in *Bacteroidota*. Similarly, Tang et al. [53] identified specific bacterial genera, including *Ignatzschineria*, *Providencia*, *Wohlfahrtiimonas*, *Proteus*, *Myroides*, and *Bacteroides*, that contribute to the growth of *S. peregrina* in response to ciprofloxacin exposure. Li et al. [86] showed that exposure to Cd disrupted both bacterial and fungal communities in the gut of honey bees, notably decreasing *Proteobacteria* and *Bacteroidota* while increasing *Firmicutes* and *Actinobacteriota*. These shifts suggest that xenobiotics can profoundly influence microbial structure and function, with downstream effects on host physiology. Therefore, microbiomic profiling not only enhances our understanding of how xenobiotics perturb insect gut ecosystems but also serves as a complementary tool for toxicological analysis, offering indirect biomarkers of exposure and effects on larval development.

## 4. Potential Applications of Machine Learning Methods in Multi-Omics Data Analysis

The integration of machine learning (ML) methods into multi-omics data analysis is rapidly gaining prominence in forensic science [96]. The rising availability of complex, high-dimensional datasets captures the physiological responses of insects to xenobiotics. Multi-omics approaches enable the identification of novel biomarkers associated with toxic exposure, thus enhancing the detection of toxic effects on insect development at various biological scales [97]. ML algorithms, such as random forests, support vector machines, and deep neural networks, offer robust capabilities for managing and interpreting these large datasets, which are particularly useful for modeling complex, nonlinear relationships between toxicological variables and development in insects [98]. For example, Li et al. [26] combined metabolomic profiling, protein microarray electrophoresis, and Fourier-transform infrared (FTIR) spectroscopy with artificial intelligence techniques to accurately estimate PMI based on death-associated biological changes. In addition, ML can be trained to identify toxin-specific gene expression profiles enabling predictions of insect developmental delays or accelerations in toxic environments [99]. Furthermore, ML can assess microbiome dynamics, detecting community shifts in gut- or cadaver-associated microbes in response to xenobiotic exposure [100]. Correlating such microbial fluctuations with insect development enhances our understanding of decomposition ecology and exposure duration, further refining PMI models. Additionally, ML algorithms can incorporate environmental variables such as temperature, humidity, and geographical location, which significantly influence insect development and succession patterns [101]. This approach allows us to simulate real-world scenarios more accurately and generate highly specific predictions [102]. Furthermore, as different necrophagous species exhibit species-specific sensitivities to various xenobiotics, ML can handle these effects in multispecies datasets [103]. This capability is critical, as mixed-species colonization is common in forensic cases and can confound PMI interpretation if species-specific xenobiotic responses are not adequately modeled.

## 5. Conclusions

Forensic entomotoxicology has emerged as a powerful alternative in cases where traditional toxicological approaches are limited, particularly when samples are no longer available due to advanced decomposition. By analyzing necrophagous insects that colonize corpses, this method enables the detection of drugs and toxicants, simultaneously evaluates their physiological impact on insect development, both of which are critical for accurate PMI estimation. Recent advancements in multi-omics technologies have provided new insights into the physiological and biochemical responses of insects to toxic exposure. These tools contribute to elucidating the molecular mechanisms underlying xenobiotic-induced developmental changes and identify reliable biomarkers of exposure. However, significant challenges remain, including species-specific variability in xenobiotic absorption and metabolism, environmental variability, and the lack of standardized protocols for insect sampling, preservation, and analysis. Furthermore, the application of ML algorithms to analyze large entomotoxicological datasets has the potential to greatly enhance the accuracy and efficiency of both xenobiotic detection and PMI estimation. In a word, forensic entomotoxicology provides a robust framework for resolving the complexities of xenobiotic-influenced decomposition.

## Figures and Tables

**Table 1:** Effects of drugs and toxins on development of necrophagous flies and their detection.

Drugs/Toxins	Dose	Food Source	Analytical Methods	Sample Preparation for Toxicological Analysis	Species of Flies	Developmental Rate	References
Methamphetamine	10 mg/kg	Kangaroo mice	HPLC–UV	The solution was extracted with CH_2_Cl_2_, and the combined organic layers were concentrated to produce p-hydroxyamphetamine. Boc_2_O was added. CH_2_Cl_2_ was then added, and the organic layer was extracted.	*Calliphora stygia* (Fabricuis, 1781)	Larval growth significantly accelerated and increased the size of all life stages. The pupal stage was prolonged 78 h.	[8]
Methamphetamine	180 mg/kg	Rabbits	GC–MS	The larvae were placed in falcon tubes, and dichloromethane was added. The tube was stored at 4 °C for 48 h to fully dissolve the matrix. The salting-out effect was attained by adding NaCl.	*Calliphora* (*Aldrichina*) *grahami* Aldrich, 1930	The developmental time to reach the pupal stage was slower. the mean length of larvae was longer.	[37]
Cocaine	17 mg/kg	Rabbit liver	GC–MS	Liver was homogenised using methanol. The supernatant was recovered, and 1 N acetic acid was added. Ether: hexane was then added. The organic layers were taken to waste and the aqueous phases were pipetted onto cation-exchange resin columns.	*Chrysomya albiceps* (Wiedemann, 1819), *Chrysomya putoria* (Wiedemann, 1830)	The larvae developed faster than the control, indicating that the drug influences and stimulates larval growth.	[38]
Morphine	3/6/12 mg/kg	Rabbits	RIA kit	Cleaned larvae were dried, weighed, and cut with scissors. To determine morphine, samples were homogenized and centrifuged for 15 min at 10,000 rpm, and the supernatant was obtained.	*Lucilia sericata* (Meigen, 1826), *Chrysomya megacephala* (Fabricius, 1794), *Sarcophaga argyrostoma* (Robineau-Desvoidy, 1830), *C. albiceps*	After morphine treatment, the larval age estimates based on the mean body length had large errors, which were 24, 27, 6, and 21 h, respectively.	[39]
Methadone	4 mg/g	Beef heart	UPLC–MS/MS	Samples were ground to powder and transferred to a glass vial after addition of deionized water and saturated ammonium chloride buffer. Following centrifugation, the clear organic phase was transferred to a clean vial and evaporated to dryness in a vacuum centrifuge.	*L. sericata*	Development seemed to be slightly decelerated in presence of high methadone concentration.	[40]
Carbamazepine and clobazam	3271.57/414.08 mg/kg	Rabbits	UHPLC/QTOF-MS	Homogenates were inserted in tubes containing methanol. Three successive stirrings were carried out to dissolve the solutes in methanol and facilitate the extraction. Then, the mixtures were centrifuged.	*Lucilia silvarum* (Meigen, 1826), *C. albiceps*, *L. sericata*	*C. albiceps* larvae fed on drugs developed faster, while the development of *L. sericata* and *L. silvarum* larvae slowed.	[34]
Malathion	1530 mg/kg	Rabbits	GC-MC	Larvae were washed and homogenized with anhydrous sodium sulfate. Acetone was added to the sample. Sodium sulfate and dichloromethane were added to the extraction and homogenized. The underlayer was collected, and the residue was re-extracted with dichloromethane.	*C. megacephala*	The maximum length of larvae and weight of pupae were observed under increasing concentrations. The rate of development varies from 12 to 36 h.	[41]
Malathion	/	/	HPLC-DAD	60 mL of acetone was added to the tested half and agitated for 45 min; the resulting extract was filtrated and washed with acetone. The acetone extracts were mixed, and anhydride sulfate sodium was added and homogenized. Extraction was perfrormed by adding dichloromethane.	*Fannia scalaris* (Fabricius, 1794)	It reduced the larval growth rate and increased the duration of the larval stage.	[42]
Glyphosate (herbicides)	7.69 mL/kg	Pigs	/	/	*L. sericata*	The duration of the developmental stages remained unchanged, but all size parameters of the puparium were reduced.	[43]
Terbufos (organophosphate)	20 mg/kg	Rats	/	/	*Lucilia eximia* (Wiedemann, 1819), *Peckia chrysostoma* (Wiedemann, 1830)	Larvae of *L. eximia* were more active, with greater frequency of body movements and lateral contractions. Immature *P. chrysostoma* were less active, with fewer body and lateral contractions.	[44]
Dimethoate	1–4 mg/kg	Sheep liver	/	/	*Chrysomya saffranea* (Bigot, 1877); *Chrysomya rufifacies* (Macquart, 1843); *Chrysomya indiana* Walker, 1861; *C. megacephala*	Dimethoate causes a delay in development. The duration increased with an increase in concentration.	[45]
Dimethoate	1–4 mg/kg	Sheep liver	/	/	*Sarcophaga peregrina* (Robineau-Desvoidy, 1830); *Sarcophaga dux* Thomson, 1869; *Sarcophaga ruficornis* (Fabricius, 1794)	Dimethoate delays the larval, pupal, and prepupal stages of development.	[46]
Benzoylecgonine and morphine	17/34 mg/kg	Pork mince	/	/	*Calliphora vomitoria* (Linnaeus, 1758)	Cocaine shortened pupation and accelerated eclosion, and the insects less in length and weight. Heroin led to lengthier pupation, and the insects were smaller and lighter.	[9]
Zolpidem tartrate	1–4 mg/kg	Buffalo liver	/	/	*C. megacephala*, *C. saffranea*	The weight, length, and width decreased as the concentration increased. The duration of both developmental stages increased as the concentration increased.	[47]
Zolpidem tartrate	1–4 mg/kg	Buffalo liver	/	/	*S. ruficornis*	The total developmental durations were prolonged when the concentration increased.	[48]
Lorazepam	1–4 mg/kg	Beef liver	/	/	*C. rufifacies*	Length, weight, and width of larvae decreased with increased concentration of lorazepam.	[49]
Ceftriaxone and levofloxacin	28.57/3.57 mg/kg	Minced pork	/	/	*L. sericata*	The time to pupation was significantly extended, and the mortality rate increased.	[50]
	28.56/3.56 mg/kg	Minced pork	/	/	*C. vomitoria*	The maggot growth was delayed by levofloxacin but not with ceftriaxone. Pupation was delayed in both antibiotics, and mortality was reduced.	[51]
	28.57/3.57 mg/kg	Minced pork	/	/	*Calliphora (Protophormia) terraenovae* Macquart, 1851	The maggot development time was significantly decreased. The time to start pupation was significantly increased. The survivability of the maggots was improved.	[52]
Ciprofloxacin	1.33 mg/kg	Pork lung	/	/	*S. peregrina*	The length of larvae increased with higher drug concentrations, while the weight of both the pupa and adult decreased significantly.	[53]
Cadmium	6.5 mg/kg (lethal)	Rats	/	/	*C. megacephala*	Development time was prolonged at higher concentrations, and larval mortality increased with increasing concentration.	[54]
Lead and cadmium ions	/	Pork liver	/	/	*Calliphora vicina* Robineau-Desvoidy, 1830	Fly larvae exhibited reduced motor activity, along with delays in puparia formation and adult emergence.	[55]
Cadmium, zinc, copper	2 mg/kg	Chicken livers	/	/	*L. sericata*	Larval and pupal survival decreased as heavy metal concentrations increased. Pupal weight and larval length were significantly different among heavy metals and concentrations.	[56]
Ethylene glycol	28 mL/kg	Beef liver	/	/	*Lucilia cuprina* (Wiedemann, 1830) *L. sericata*	Neither species can survive in high concentrations. The developmental time of both species is slower than the control; the body length of the immatures is also smaller.	[57]

**Table 2:** The impact of xenobiotics on the succession patterns of necrophagous insects and cadaveric decomposition process.

Toxic Substances	Dose	Food Source	Location	Community Succession	Rate of Decay	References
Diazinon	100/300 mg/kg	Rabbits	Eastern Amazon	The adult specimens in the control group with the highest abundance were observed only from the advanced decay stage onward. In the dry stage, abundance was higher in control than in treated carcasses. The larvae of *C. albiceps* (76.3%), *C. putoria* (1%), and *L. eximia* (22.7%) were identified; the number of immatures was higher in control.	Diazinon slowed down the decomposition stages.	[58]
Malathion	1530 mg/kg	Rabbits	Sun Yat-Sen University, Guangzhou	*C. megacephala* was the most abundant adult species in all groups. Larvae of *C. rufifacies* were only collected from the control; the appearance of beetles on the treated carcass was later by 1 to 3 days than on the control carcass.	Malathion altered decomposition rates.	[41]
Terbufos	5/10 mg/kg	Rats	Federal University of Rio Grande do Norte	*C. albiceps* was collected with a clearly high dose of terbufos; *L. eximi*a *S. nudiseta* and *P. chrysostoma* were collected with low doses.	Higher doses accelerated decomposition.	[59]
Thiamethoxam	/	Pigs	Dourados, Mato Grosso do Sul, Brazil	1462 specimens from Diptera and 279 Coleoptera were identified, mainly including *C. megacephala* and *L. eximia*. In the control group, 641 Diptera and 385 Coleoptera were collected, obtaining *C. putoria*, *L. cuprina*, *C. albiceps*, and *D. maculatus*.	The experimental group took longer to reach the late stage of corruption than the control group.	[60]
Aluminum phosphide (AIP)	27.4 mg/kg	Rabbits	Beheira, Egypt	*C. rufifacies* was detected only in control. *C. albiceps* and *C. megacephalla* were presented in all groups but exhibited variations across the decomposition process.	AlP appeared to delay the decomposition process.	[61]
Atrazine	3000 mg/kg	Rats	Zagazig University, Egypt	A delay in the colonization of insect fauna was observed in treatment. In the control group, Dipteran insects were the most dominant insects (57.14%), followed by Coleopteran insects (42.85%). The treatment showed 42.85% for insects of order Diptera and 57.14% for Coleoptera.	Decay of carrions was delayed in treatment.	[62]
Heroin	6/12/18 mg	Rabbits	Riyadh, Saudi Arabia	Heroin did not have a significant impact on the number of insects, but during the summer, M. domestica and C. albiceps were more attracted to treated carcasses with a higher dose. Flies were more attracted to carcasses with a higher dose.	Heroin appeared to delay the decomposition process.	[63]
Alcohol	25/50/75 mL	Rabbits	Riyadh, Saudi Arabia	Alcoholic beverages did not significantly affect insect succession patterns.	The treated rabbits took two days longer than the untreated ones to reach the dry stage in winter and one day longer in summer.	[64]
Lead (Pb)	0.18/0.2 mg/kg	Pigs	Lagos State University, Nigeria	The decomposition rate of pigs fed with lead-contaminated feed attracted insects. *C. chloropyga* was the most predominant.	The decomposition rate of pigs fed with lead-contaminated feed increased the rate of hair fall.	[65]

## Data Availability

No new data were created or analyzed in this study. Data sharing is not applicable to this article.
